# Reliability and validity of the Shona version of the Exercise Benefits and Barriers Scale in Zimbabwean adult people living with HIV/AIDS

**DOI:** 10.3389/fpsyt.2023.1188689

**Published:** 2023-08-24

**Authors:** Jermaine M. Dambi, Ben Domingue, Melanie Abas, Dixon Chibanda, Tonya M. Esterhuizen

**Affiliations:** ^1^Rehabilitation Sciences Department, Faculty of Medicine and Health Sciences, University of Zimbabwe, Harare, Zimbabwe; ^2^Division of Epidemiology and Biostatistics, Department of Global Health, Faculty of Medicine and Health Sciences, Stellenbosch University, Tygerberg, South Africa; ^3^Friendship Bench, Harare, Zimbabwe; ^4^Graduate School of Education, Stanford University, Stanford, CA, United States; ^5^Health Service and Population Research Department, Institute of Psychiatry, Psychology and Neuroscience, King’s College London, London, United Kingdom; ^6^Mental Health Unit, Faculty of Medicine and Health Sciences, University of Zimbabwe, Harare, Zimbabwe; ^7^Department of Population Health, Faculty of Epidemiology and Population Health, London School of Hygiene and Tropical Medicine, London, United Kingdom

**Keywords:** Exercise Benefits and Barriers Scale, translation, validity, reliability, HIV

## Abstract

**Introduction:**

Despite the widely known benefits of physical activity (PA), only 25% of people living with HIV (PLHIV) meet the WHO-recommended minimum PA levels. Consequently, it is essential to understand PA barriers and facilitators using objective measures. Although the Exercise Benefits and Barriers Scale (EBBS) is extensively used, its psychometric evidence is fragmented and has not been previously validated in PLHIV. This study aimed to translate and validate the EBBS Shona version in Zimbabwean PLHIV.

**Methods:**

A cross-sectional study was used to recruit 567 PLHIV from four (4/9) randomly selected polyclinics (primary healthcare facilities) in urban Harare, Zimbabwe. We recruited adult patients (aged ≥18 years) with a confirmed diagnosis of HIV. Participants had to be willing to provide informed consent, not acutely unwell, and proficient in the Shona language. We used a forward-backwards translation method to translate the EBBS from English to Shona, a native Zimbabwean language. After cross-cultural adaptation, we pretested the draft version in 10 PLHIV to assess the face validity, understandability and cultural appropriateness using semi-structured interviews. Thereafter, the EBBS was administered to 567 consecutively-selected PLHIV. Factor analyses were performed for construct validity evaluation.

**Results:**

Most participants were female (72.5%) and reached secondary/high school (78.8%), with a mean age of 39.9 (SD 12.1) years. The EBBS-Shona version yielded a four-factor solution consisting of three benefits factors and one barrier factor against the originally postulated six-factor structure. The EBBS-Shona yielded α = 0.85 and intraclass correlation coefficient = 0.86, demonstrating excellent reliability. Increased perception of exercise benefits was positively correlated with increased reports of physical activity, higher health-related quality of life and lower psychiatric morbidity; evidence for construct validity.

**Discussion:**

This study demonstrates the validity and reliability of the EBBS-Shona version in Zimbabwean PLHIV. The EBBS-Shona version can be used for research and clinical purposes to glean data to inform the development, implementation, and evaluation of bespoke PA interventions for PLHIV.

## 1. Introduction

The Sub-Saharan Africa (SSA) region has the world’s highest burden of HIV/AIDS (1). A concurrent high burden of non-communicable diseases (NCDs), including common mental health disorders (CMDs) in people living with HIV/AIDS (PLHIV), is obstructive to efforts to eradicate the HIV/AIDS epidemic (1, 2). For example, depression is thrice as common in PLHIV compared to the general population (3, 4). Poor mental health is regrettably associated with; high morbidity and mortality, high treatment costs, lower economic productivity, increased disability, and lower health-related quality of life (HRQoL) (4–8). Mental healthcare in PLHIV is traditionally mainly offered as pharmacotherapy and psychotherapy, with evolving evidence supporting the effectiveness of complementary interventions, including physical-activity-based regimens (9, 10). However, not all patients managed through pharmacotherapy and psychotherapy get into remission. Also, these core methods neither address the physical problems (e.g., loss in muscle strength, fatigue) associated with CMDs in PLHIV (9, 10). Furthermore, there is a vast mental health treatment gap in low-middle-income countries (LMICs) due to capital and human resource shortages (11, 12). For example, over 75% of PLHIV in LMICs with CMDs do not have equitable access to mental healthcare (11). There is a greater call to consider complementary treatment strategies, such as physical activity (PA), to close the mental health treatment gap in PLHIV (9, 10).

Physical activity is a low-hanging fruit as it is a cost-effective and scalable intervention for managing CMDs in PLHIV (12–14). Despite the demonstrated benefits of physical activity (PA), physical inactivity remains a global epidemic (15, 16). Nearly 30% of the global adult population (≈1.4 billion adults) are physically inactive and at high risk of NCDs (17). The burden of physical inactivity in PLHIV is even more significant, with only 25% of PLHIV meeting the minimum recommended PA levels (10, 18). Further, PLHIV people with CMDs are less likely to engage in PA and exhibit sedentary behaviour, creating a vicious cycle of NCDs risk (18, 19). The high prevalence of bodily pain, depression, HIV-related stigma, lack of social support, and opportunistic infections are salient predictors of physical inactivity in PLHIV (18, 19). Given the high burden of NCDs, the World Health Organization has put forward an ambitious target to decrease physical inactivity by 15% by 2030 as part of a holistic plan to curtail the burden of NCDs (20). The action plan recommends bespoke PA interventions targeting high-risk populations, including PLHIV (17, 20). More so, PA is essential in preventing and treating most NCDs (10, 14). A recent meta-analysis shows the effectiveness of PA in managing depression and anxiety in PLHIV, with large effects [SMD −0.84 (CI: −1.57; −0.011)] (14). Engagement in PA by PLHIV is associated with; increased CD4 count, improved cardiovascular fitness and endurance, lower blood pressure, improved self-esteem and body image, social connectedness, and decreased premature mortality, among many benefits (9, 10, 13, 14, 19). Taken together, PA engagement by PLHIV optimises; immune functioning, mental and physical health, social outcomes, economic productivity, and overall improvement in HRQoL (9, 10, 12–14, 19).

Promoting and implementing PA interventions is essential, particularly in the SSA region, which faces a dual, high burden of HIV/AIDS and NCDs (10). Unfortunately, physical activity is not integral to most HIV rehabilitation programs in SSA, with dropout rates as high as 30% (10). With the need to promote PA, there is a need to objectively measure PA, including understanding context-specific barriers, facilitators, and general awareness of the importance of PA (10, 12, 21). Perceived benefits and barriers to exercise are salient to PA engagement patterns (22). Importantly, there is a stern need to understand the psychosocial, socioeconomic, ecological and policy-related factors influencing PLHIV engagement in PA using validated, multidimensional outcome measures (10). One commonly used measure is the Exercise Benefits and Barriers Scale (EBBS) (12). Developed in the Unted States, the original EBBS has 43 items; 29 items measure the benefits of exercise, with 14 measuring barriers. The EBBS was initially validated in university students. It yielded a nine-factor solution (five benefits and four barriers factors) that accounted for 67.1% of the variance and yielded a Cronbach’s alpha of 0.74, showing adequate structural validity and reliability, respectively (23). Over decades, the EBBS has been extensively applied globally and has been translated and adapted in Iran (24, 25), Brazil (26), Malaysia (27), Mexico (28), and Turkey (29). However, its transcultural validity and reliability evidence are fragmented. Extensive use does not necessarily equate to psychometric robustness; this may cause inaccurate comparisons and conclusions. More so, follow-up validation studies have yielded differential factorial solutions, with some yielding 10- and 7-factor solutions (22, 30). To improve the EBBS psychometric performance, Koehn and Amiradollahian applied hierarchical confirmatory factor analysis to develop a concise six-factor, 26-item shortened version (22). The shortened version (EBBS-SF) was validated in 565 United Kingdom university students. It yielded excellent psychometric properties and can be of greater utility for routine research and clinical use due to its brevity (22) compared to the original 43-item version (23). However, it is essential to formally adapt and validate the EBBS-SF before use in different contexts. Also, it is paramount to apply robust translation methods to attain semantically and conceptually-equivalent language versions (31). Therefore, this study aimed to translate and validate the EBBS into Shona, a Zimbabwean native language. This study specifically assessed the structural validity, internal consistency, construct validity, known-group validity, and test re-test reliability of the EBBS-Shona version in PLHIV. There is a dearth of data on standardized, validated and culturally sensitive measures of PA in this population.

## 2. Materials and methods

### 2.1. Study setting

Participants were recruited from four randomly selected urban polyclinics (4/9) managed by the City of Harare Health Department. Zimbabwean public urban healthcare is structured into a three-tier system. Family healthcare facilities are the basic entry-level, with polyclinics and central hospitals constituting the second-and third-tier levels. HIV services, such as testing and pharmacotherapy, are provided at all primary healthcare facilities. There are dedicated HIV care clinics at all primary care facilities.

### 2.2. Study design

This study was done in two phases: the first stage involved the translation and adaptation of the EBBS into Shona. We utilised a forward-backwards translation method (31). First, two translators independently translated the EBBS from English to Shona. The forward translation was reconciled into one consolidated version through a panel discussion between the translators, the research assistant, and the researcher. Emphasis was on the attainment of a colloquial and conceptually-equivalent translation. After that, another set of two independent translators blindly back-translated the Shona version into English. Again, a second panel was convened between the translators and the research team. The emphasis was the attainment of a translation with literal and conceptual meaning. Third, the backward translation was compared against the original version through a panel discussion. We utilised professional and bilingual translators, i.e., they were all proficient in English and Shona. Last, cognitive debriefing interviews were performed by administering the EBBS-Shona version to 10 PLHIV to identify any problematic items and assess the understandability of the translation. Appropriate changes to the Shona version were made according to the feedback received from PLHIV. The EBBS-Shona version was validated using a cross-sectional design in the second phase.

### 2.3. Participants

We recruited adult patients (aged ≥18 years) proficient in Shona and with a confirmed diagnosis of HIV, according to doctors/clinician notes. Participants acutely unwell and/or requiring emergency treatment, with cognitive impairments and or in an intoxicated state on the day of data collection were excluded. Trained research assistants (Physiotherapy and Occupational Therapy graduate trainees) subjectively assessed the prospective participants’ mental state as indicated by the coherence in responses. Where appropriate, the Mini-Mental State Examination (cut-off point ≤23) was utilised to quickly assess a participant’s mental status (32).

### 2.4. Sample size calculation

According to Schmidt et al., the recommended recruitment ratio for confirmatory factory analysis is 5–20 candidates per item (33). Applying a 10:1 participant-to-item ratio, we set to recruit at least 260 participants. We doubled the minimum sample size to ensure two equal datasets for exploratory and confirmatory factor analysis. To evaluate the EBBS-Shona version test–retest reliability, we collected data from 50 randomly selected participants at baseline and after a fortnight per COnsensus-based Standards for the selection of health Measurement INstruments (COSMIN) guidelines (34).

### 2.5. Sampling

Harare polyclinics were first stratified according to the three socioeconomic zones, i.e., low-, medium- and high-density strata. A polyclinic(s) was randomly selected from each of the strata. *A priori* ratio of 1:1:2 was used to select participants according to socioeconomic status proportionally. The stratification resulted in participants from high-density suburbs being selected more; they were 50% of the study population. Consecutive sampling was used to select cases.

### 2.6. Data collection

Prospective participants were recruited consecutively as they presented for routine HIV care. On the day, trained research assistants first sensitised patients regarding study procedures and rights. Sensitisation was done as prospective participants waited to receive care in the treatment waiting areas. Participants meeting the inclusion criteria and interested in participating were taken to private spaces reserved for the study to provide written consent. The study questionnaires were interviewer or self-administered depending on the participants’ literacy level or preference for the data collection method. Based on the previous fieldwork, we set the threshold of self-completion to at least secondary education, i.e., 9 years from kindergarten education as a minimum. However, there are instances where participants opted for interviewer-administered mode, for instance, due to impairment, such as bodily pain.

### 2.7. Measures

The primary outcome measure was scores on the EBBS-Shona short version. We also collected secondary data, i.e., HRQOL, anxiety/depression, and physical activity.

- EEBS-SF – the EBBS-SF has 26 items: 19 measure the benefits of exercise, with seven measuring barriers. Perceived benefits of PA are classified into these four domains, i.e., life enhancement (LE), physical performance (PP), psychological outlook (PO), and social interaction (SI). Barriers are classified as facilities access (FA) or time expenditure (TE). The items on the EBBS are measured on a four-point Likert scale ranging from strongly disagree = 1 to strongly agree = 4. The scores range for the benefits and barrier domains are 4–76 and 4–28, respectively (22).
- EQ-5D 5 L – the EQ-5D is a generic, self-report HRQoL outcome measure. Using a five-point scale, respondents rate challenges with; mobility, self-care, usual, pain/discomfort, anxiety, and depression. The Shona version is validated, and normative utility scores are available for the Zimbabwean population (35).
- PHQ-4 – the PHQ-4 is a brief anxiety/depression screener. Respondents rate the frequency of experience of the enlisted anxiety/depressive symptoms in the previous 2 weeks on a four-point Likert scale ranging from “not at all = 0” to “all the time = 3” to give a cumulative score of 0–12. The PHQ-4 has been extensively used for clinical and research purposes and validated in PLHIV in the research setting (36).
- International physical activity questionnaire short form (IPAQ-SF) – the IPAQ-SF is an extensively used and psychometrically-robust seven-item PA measure (37). It assesses PA under the following four levels of intensity: vigorous-intensity activity (e.g., aerobics), moderate-intensity activity (e.g., leisure cycling), walking and sitting (37).
- The sociodemographic questionnaire extracted participants’ demographics, i.e., age, gender, education level, employment status, marital status, and financial status.

### 2.8. Data analysis

Descriptive statistics (e.g., frequencies, means) were used to describe participants’ characteristics and study outcome measures. Data were randomly split into two for exploratory and confirmatory factor analysis to evaluate the EBBS-Shona version’s structural validity – see Supplementary File S1 for the detailed analysis plan. Further, reliability as internal consistency and test–retest reliability were evaluated using Cronbach’s Alpha (criteria: α ≥ 0.7) and the Intraclass Correlation Coefficient (criteria: ICC ≥ 0.4), respectively. The Pearson correlation coefficient was applied to evaluate the construct validity by determining the correlation between EBBS-Shona scores and secondary outcome measures (e.g., depression); criteria, *r* ≥ 0.4. Known-group validity was assessed using t-tests by assessing differences in EBBS-Shona scores across gender. All analyses were done at α = 0.05 using SPSS (Version 28) and Stata (Version 17).

### 2.9. Ethical considerations

Ethical approval for the study was granted by the Stellenbosch University Health Research Ethics Committee (Ref: S22/06/111) and the Medical Research Council of Zimbabwe (Ref: MRCZ/B/2397).

### 2.10. Patient and public involvement

Participants interpreted the EBBS-Shona alpha version during the cognitive debriefing stage to assess the understandability and appropriateness of the translation.

## 3. Results

### 3.1. Participants’ characteristics

Most participants were female (72.5%), reached secondary/high school education (78.8%), married (51.1%), informally employed (33.2%), and reported inadequate finances (72.6%). The participants’ mean age was 39.9 (SD 12.1) years (Table 1).

**Table 1:** Study participants’ characteristics, *N* = 567.

Variable	Attribute	Frequency, *n* (%)
Gender	Female	411 (72.5)
	Male	156 (27.5)
*Age	Mean (SD)	39.9 (SD 12.1)
Educational status	Primary	67 (11.8)
	Secondary/High	447 (78.8)
	Tertiary	53 (9.3)
Relationship status	Currently married	290 (51.1)
	In a relationship	67 (11.8)
	Separated/divorced	83 (14.6)
	Widowed	70 (12.3)
	Not in a relationship	57 (10.1)
Employment status	Unemployed	121 (21.3)
	Informally employed	188 (33.2)
	Formally employed	126 (22.2)
	Housewife	92 (16.2)
	Other	40 (7.1)
Financial situation	Very inadequate	179 (31.6)
	Inadequate	233 (41.1)
	Neutral	133 (23.5)
	Adequate	18 (3.2)
	Very adequate	4 (0.7)

* Data not presented in *n* (%) format.

### 3.2. Exploratory factor analysis

There were spread responses on the EBBS, with the barriers sub-scale having the lowest means compared to the benefits sub-scale (Supplementary Table S2). Data were factorable given adequate sampling adequacy, i.e., Kaiser–Meyer–Oklin (KMO) =0.918 and 0.806, for benefits and barriers, respectively and statistically significant Bartlett Tests of Sphericity (*p* < 0.001). Items correlated reasonably with items within the same scale, with few correlations <0.3, and there was no multicollinearity. Except for the psychological outlook scale, the item-total correlation (ITC) range across the factors was reasonable in the range 0.575–0.746; Supplementary Table S3. The Kaiser criterion (Supplementary Table S4) and an inspection of the structure and pattern matrices (Table 2) supported the retention of one and three factors for barriers and benefits, respectively. Multiple cross-loadings were prevalent for factors one and two after Promax (oblique) rotation. The four factors accounted for 71.1% accumulative variance.

**Table 2:** EBSS-SF pattern and structure matrices.

Benefits items							
Pattern Matrix				Structure Matrix			
Item	Factor 1	Factor 2	Factor 3	Item	Factor 1	Factor 2	Factor 3
EBBS23	0.820			EBBS19	0.804	0.628	
EBBS18	0.796			EBBS18	0.758	0.564	
EBBS19	0.781			EBBS13	0.745	0.669	
EBBS22	0.595			EBBS20	0.691	0.626	
EBBS13	0.568			EBBS15	0.678	0.636	
EBBS20	0.511			EBBS22	0.677	0.560	
EBBS15	0.466			EBBS7	0.668	0.652	
EBBS7	0.406	0.340		EBBS23	0.661	0.422	
EBBS9		0.827		EBBS9	0.534	0.750	
EBBS5		0.749		EBBS12	0.676	0.744	
EBBS11		0.619		EBBS24	0.683	0.739	
EBBS12		0.549		EBBS11	0.596	0.712	
EBBS24		0.524		EBBS26	0.618	0.649	
EBBS26		0.423		EBBS5	0.409	0.622	
EBBS17		0.340		EBBS17	0.522	0.540	
EBBS2			0.895	EBBS2			0.899
EBBS3			0.715	EBBS3			0.712
EBBS1			0.619	EBBS1			0.619
EBBS6			0.592	EBBS6			0.588
**Barriers items**							
**Item**	**Factor 1**						
EBBS14	0.760						
EBBS8	0.619						
EBBS21	0.587						
EBBS10	0.530						
EBBS4	0.482						
EBBS25	0.473						
EBBS16	0.429						
EBBS14	0.760						

### 3.3. Confirmatory factor analysis

**Figure 1:**
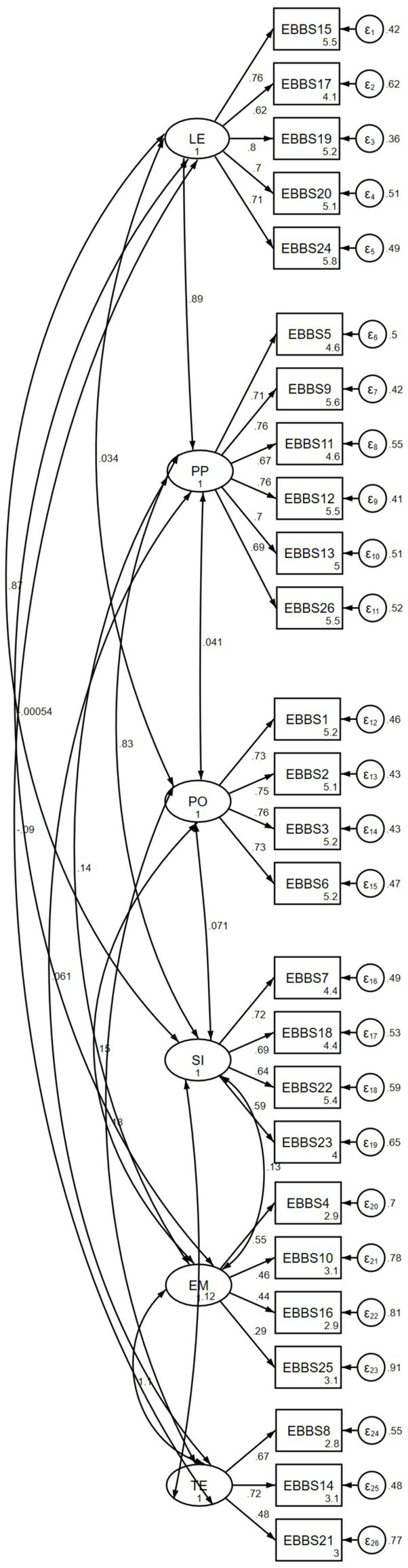
The EBBS six-factor model showing the correlations between the four benefits and two barriers factors.

Table 3 shows an assessment of the congeneric and combined scale models, including the cross-validation of the EFA solution. Assessment of the congeneric models unequivocally supports a correlated four-factor model for the benefits subscale (Supplementary Table S5). For the barriers subscale, the results were indeterminant; the one-factor and correlated two-factor models are plausible, given the mixed evidence of fit indices. For the combined scale, the correlated six-factor model showed the best fit. However, the likelihood ratio (*p* < 0.001) and the RMSEA (=0.062) showed misfit, whilst the normed chi-square test displayed slight misfit (*X*^2^/*df* = 2.1). Last, the correlated 4-factor model gleaned from EFA displayed satisfactory model fit; the model parallels the performance of the correlated six-factor model. The 6-factor model demonstrates the greatest parsimony and is illustrated in Figure 1.

**Table 3:** Comparison of the goodness of fit for the 1-, 2-, 3- and 4-factor CFA models.

	Likelihood ratio	Population error	Information criteria		Baseline comparison		Size of residuals	
Index	*χ2/df*	RMSEA (95% CI)	AIC	BIC	CFI	LFI	SRMR	SD
Criteria	Fit: *χ*^2^/*df* ≤ 2	Fit: ≤ 0.06	Fit: model with lowest AIC	Fit: model with lowest BIC	Fit: ≥0.90	Fit: ≥0.90	Fit: ≤0.06	Fit: greatest SD value
M1: Benefits one-factor model	5.2	0.122 (0.114; 0.131)	8739.1	8947.1	0.740	0.707	0.111	0.928
M2: Benefits three-factor model	2.5	0.073 (0.064; 0.083)	8325.8	8544.7	0.908	0.894	0.051	0.991
M3: Benefits uncorrelated four-factor model	5.2	0.121 (0.113; 0.130)	8728.5	8936.5	0.744	0.712	0.271	0.999
M4: Benefits correlated four-factor model	2.2	0.065 (0.056; 0.075)	8278.4	8508.2	0.928	0.916	0.048	0.996
M1: Barriers one-factor model	3.7	0.098 (0.070; 0.127)	4219.0	4295.6	0.894	0.842	0.063	0.778
M2: Barriers uncorrelated two-factor model	13.1	0.206 (0.180; 0.234)	4349.8	4426.5	0.530	0.296	0.205	0.905
M3: Barriers correlated two-factor model	3.9	0.101 (0.073; 0.131)	4219.7	4300.0	0.895	0.831	0.062	0.741
M1: Full-scale one-factor model	10.0	–	13275.9	13556.9	1.0	-	0.117	0.928
M2: Full-scale uncorrelated six-factor model	2.1	0.062 (0.055; 0.069)	12498.0	12808.2	0.891	0.878	0.062	0.999
M3: Full-scale correlated six-factor model	2.1	0.062 (0.055; 0.069)	12492.4	12831.7	0.895	0.880	0.055	0.999
M4:Full-scale uncorrelated 4-factor model	2.3	0.067 (0.060; 0.073)	12544.8	12840.4	0.874	0.861	0.063	0.998
M4: Full-scale correlated 4-factor model	2.3	0.067 (0.060; 0.073)	12544.2	12850.7	0.875	0.861	0.059	0.998

Best fit.

### 3.4. Internal consistency and test-retest reliability

Except for the barriers factors, the benefits sub-scales (α = 0.77–0.85) and summative EBBS scores (α = 0.85) collectively yielded excellent IC values (Table 4). The scale IC value did not improve by deleting any items (Supplementary Table S6), which is evidence of collective item reliability. The ICC (95% CI) for 52 participants at baseline and after 2 weeks was 0.87 (0.80:0.91); this is evidence of longitudinal stability.

**Table 4:** EBBS subscales IC values.

Scale	α	ICC (95% CI)
PO	0.81	0.81 (0.79; 0.84)
PP	0.85	0.85 (0.84; 0.87)
LE	0.77	0.77 (0.74; 0.80)
SI	0.83	0.83 (0.81; 0.85)
EM	0.5	0.50 (0.43; 0.56)
TE	0.67	0.67 (0.62; 0.72)
Benefits subscale	0.89	0.89 (0.88; 0.90)
Barriers sub-scale	0.73	0.73 (0.70; 0.77)
Scale level	0.85	0.85 (0.84; 0.87)

### 3.5. Known-group validity

Except for the psychological outlook (PO) domain, scores for all genders were comparable (Supplementary Table S7). Males were more likely to have a higher perception of the psychological benefits of exercise than females, with mean PO scores of 13.8 versus 13.1. the differences were statistically significant; *t* (*df* = 565) = 3.59, *p* < 0.001.

### 3.6. Construct validity/hypothesis testing

Increased perception of exercise benefits was poorly and positively correlated with increased reports of physical activity (*r* = 0.099; *p* = 0.019), higher HRQoL (*r* = −0.094; *p* = 0.026) and lower psychiatric morbidity (*r* = −0.118, *p* = 0.005). Last, the perceived barriers and benefits subscales were weakly and negatively correlated (*r* = −0.118; *p* = 0.005; see Supplementary Table S8).

## 4. Discussion

We set out to translate, adapt, and validate the EBBS-Shona version in Zimbabwean PLHIV. To the best of our knowledge, this is the first study to formally validate the EBBS in PLHIV. Our data provide initial evidence of psychometric robustness regarding structural-, construct- and known-group validity and reliability (internal consistency and test–retest reliability). The EBBS-Shona version yielded a four-factor solution consisting of three benefits factors and one barrier factor against the originally postulated six-factor structure (22). However, our results demonstrate a second-order factor structure with items lumped into benefits and barriers (12, 22, 24). Structural validation results were contradictory; this is not uncommon (38). Exploratory factor analysis (EFA) and confirmatory factor analysis (CFA) supported four- and six factors, respectively. Structural validity is the most essential psychometric; the rest of the psychometric properties depend on the quality thereof (34, 38, 39). Only the physical outlook (PO) factor was replicated for benefits sub-scales in EFA. The homogeneous composition of the items can partially account for the congruency; items in this factor exclusively describe/measure the mental health benefits of physical activity (22, 23). However, the PO factor exhibited the lowest item-subtotal correlations (ITC range: 0.21–0.30) against the ITC range of 0.66–0.77 for the United Kingdom validation study (22). The discrepancies could be accounted for the lack of exact Shona words for “tension,” “stress,” and “mental health”; translation of these items was challenging. The remaining three benefits sub-scales (life enhancement, physical performance, and social interaction) loaded onto two distinct factors with several items cross-loading onto the two factors. The high prevalence of cross-loadings across the two factors could reflect cultural differences and perceptions of items under these factors.

Unlike the postulated two-factor solution, our outcomes support a one-factor solution for perceived barriers. This implies that participants could not differentiate between access-related and time-related barriers to physical activity. The high factor loadings and item-total correlations for the barriers sub-scale further support the one-factor solution. Again, cultural differences and a lack of linguistic diversity in the target language could account for the disparity. The Shona language has a limited vocabulary compared to English (40); this is potentially reflected in the conceptualisation of barrier-related factors as a solitary factor, as some of the words signifying different concepts were used interchangeably.

As for CFA, both four- and six-factor solutions are plausible, with the six-factor model being the most robust. However, for both models, the likelihood ratio showed a misfit, the normed chi-square test displayed a slight misfit, and the RMSEA showed evidence of misfit; these results are similar to the Persian and Mexican versions validation studies (24, 28). The likelihood ratio is prone to misfitting in large sample sizes, i.e., samples ≥200 (41); we analysed data for 283 participants. The normed Chi-square is considered a befitting alternative for large samples (38, 41); again, it showed a slight misfit. The RMSEA is a robust absolute fit index for CFA model estimation; it showed a model misfit. Normality violation could have resulted in model misspecification (42). Model misfits are prevalent in CFA due to the stringent assumption that an item must load onto one latent factor (38, 41), which may be impractical when analysing latent constructs such as perceived barriers and benefits. The strict assumption can lead to parameter estimation bias (38, 41). Multiple cross-loadings in benefits items during EFA testify to the potential failings of CFA assumptions (38). Collectively, the misfitting indices may imply a further need for improvements in the EBBS to increase factorial structure robustness. It may also be needful to apply other psychometric evaluation techniques, such as item response theory and exploratory structural equation modelling (ESEM), to overcome CFA limitations. For instance, in ESEM, cross-loadings across factors are permissible; this may yield a more parsimonious solution (38).

If EFA and CFA results are indifferent, CFA outputs usually take precedence (38). Here, mathematically, it was plausible to accept the six-factor model per CFA outputs. However, structural validation is an “art,” and there is a need to consider both the statistical outputs and a qualitative underpinning of the factors, including the understandability and interpretability of the factor solution (38, 41). Given the importance of model parsimony (42), it seems reasonable to adopt the four-factor solution of the EBBS-Shona version. More important, the EFA solution was succinct, accounting for 71.1% of the total variance, demonstrating the adequacy of the solution in measuring benefits and barriers to PA engagement. Also, the fit indices for the four-factor solution were marginally different from the six-factor solution and were within and or above the minimum set criteria (38, 41, 42). Nevertheless, the divergent factor analysis solutions have two-fold implications. First, the perceived barriers and benefits sub-scale should be interpreted separately, as the summative/combined score does not have a mathematically “intuitive” meaning. Second, there is a strong need to expand the content validity of the barriers scale; this may subsequently improve the factorial validity.

The EBBS-Shona is reliable, as evidenced by a high internal consistency at the scale level; this is comparable to previous studies, which also yielded high IC values between 0.67 and 0.83 (22, 25, 30). At the factor level, the two distinct barriers factors had sub-par IC levels. However, the one-factor solution was marginally above the minimum set criteria value (α = 0.72) (42). The lower IC values of the barriers items are unsurprising and comparable to other studies (22, 25). For example, the PO factor yielded α = 0.58 in an Iranian translation and validation study (25). Internal consistency values are a function of item numbers. Generally, the more items a factor consists of, the more it is likely to yield high reliability indices (43). The barriers scales have fewer items than the benefits scale (7 vs. 19), hence the anticipated discrepancies in IC values. There may be a need to increase the number of items to increase the construct and content validity of the barriers factor (22, 30). Still, the high IC values also support the robustness of the one-factor solution for barrier items. Also, similar to other studies (25, 29), our data show the longitudinal stability of the EBBS, as evidenced by high intraclass correlation coefficient values. It is doubtful that participants’ perceptions of the benefits and barriers could have changed drastically within 2 weeks. Collectively, all items on the EBBS-Shona consistently measured the same construct(s) within the 2 weeks.

We also tested the construct validity by assessing the correlations between EBBS scores and secondary outcome measures. Increased perception of exercise benefits was positively correlated with increased reports of physical activity, higher HRQoL, and lower psychiatric morbidity. This is theoretically plausible as previous studies have shown that higher perceived benefits are linked to increased PA engagement per the health beliefs model (10). The increased PA subsequently leads to improved mental health outcomes and HRQoL (10, 22). Conversely, more significant barriers were associated with low PA and a myriad of negative physical and psychosocial indices (9, 22). Also, our results concur with the health beliefs model regarding the negative correlation between perceived barriers and benefits (22, 30). Except for the psychological outlook sub-scale (PO), our findings do not seem to support gender differences in the perception of barriers and benefits; this follows previous studies (22, 30). A previous study postulated that males are likelier to have high perceptions of the recreational and social benefits of exercising than females (30); this may account for the higher PO scores in the current study. However, the low inter-item correlations for the PO sub-scale may have led to a spurious statistical finding; the factor may have unstable performance. Altogether, the EBBS equally performed across the genders, and scores can be used for direct comparison.

### 4.1. Study strengths and limitations

Study strengths include applying a robust translation process and using both EFA and CFA to test the EBBS-Shona dimensionality. This study builds upon the methodological limitations of previous studies. First, we used the generalised least squares, common factor analysis method (39). Other studies have incorrectly applied principal component analysis, which is not a true factor analysis method (23, 25, 28). Unlike previous studies that used orthogonal rotation (25, 28, 30), we applied oblique rotation to enhance factors’ interpretability in EFA. Orthogonal rotation must be used in rotating uncorrelated factors, a rare occurrence in behavioural sciences (39). Orthogonal rotation is inappropriate for the EBBS, given the negative correlation between perceived barriers and benefits (22, 30). Third, we used electronic data collection, including implementing mandatory responses and skip logic patterns which negate missing values, an essential methodological consideration for robust factor analysis outputs (43). However, facility-based, cross-sectional studies are prone to non-respondent selection and recall biases. For instance, our sample was gender-biased, with very few men (27.5%); our findings may have limited applicability. Future studies need to optimise the recruitment of men by applying stratified random sampling. The study may not be generalisable to PLHIV living outside Harare. Still, as the capital city, most people migrate to Harare for several reasons, including seeking jobs, so the sample will probably be reasonably representative given the study’s large sample. We recruited participants using consecutive sampling. Ideally, participants should have been recruited using random sampling, a requirement for factor analyses. However, random sampling was not feasible as participants arrived at different intervals, and the reduced patient volumes during the data collection could have prolonged the data collection period beyond the limits of the available study budget.

## 5. Conclusions and future directions

This study demonstrates the validity and reliability of the EBBS-Shona version in Zimbabwean PLHIV. The EBBS-Shona version can be used for research and clinical purposes to glean data to inform the development, implementation, and evaluation of bespoke PA interventions for PLHIV. Also, the EBBS could be applicable to other conditions or general populations in persons living in low-resource settings such as Sub-Saharan Africa, given the potential commonality in the need to increase populations’ physical activity. Also, barriers to PA engagement (e.g., lack of safe environment and equipment) are likely to be universal, hence the potential utility of the EBBS in non-HIV populations. Future studies are needed for continuous psychometric evaluations and gleaning normative data.

## Data availability statement

The raw data supporting the conclusions of this article will be made available by the authors, without undue reservation.

## Ethics statement

Ethical approval for the study was granted by the Stellenbosch University Health Research Ethics Committee (Ref: S22/06/111) and the Medical Research Council of Zimbabwe (Ref: MRCZ/B/2397). The patients/participants provided their written informed consent to participate in this study.

## Author contributions

JD was primarily responsible for study conceptualisation, protocol writing, data collection, data analysis and drafting the first full version of the manuscript. TE was JD’s primary supervisor for the minor dissertation for the MSc Clinical Epidemiology program at Stellenbosch University. BD, MA, DC, and TE conceptualised the study and edited all versions of the manuscript. All authors contributed to the article and approved the submitted version.

## Conflict of interest

The authors declare that the research was conducted in the absence of any commercial or financial relationships that could be construed as a potential conflict of interest.

## Publisher’s note

All claims expressed in this article are solely those of the authors and do not necessarily represent those of their affiliated organizations, or those of the publisher, the editors and the reviewers. Any product that may be evaluated in this article, or claim that may be made by its manufacturer, is not guaranteed or endorsed by the publisher.

## Supplementary material

The Supplementary material for this article can be found online at: https://www.frontiersin.org/articles/10.3389/fpsyt.2023.1188689/full#supplementary-material

## Abbreviations

AIC: Akaike’s information criterion
BIC: Bayesian information criterion
CFA: confirmatory factor analysis
CFI: comparative fit index
CMDs: common mental health disorders
COSMIN: COnsensus-based Standards for the selection of health Measurement Instruments
EBBS: Exercise Benefits and Barriers Scale
EBBS-SF: Exercise Benefits and Barriers Scale Short Form
EFA: exploratory factor analysis
EQ-5D: EuroQol- 5 Dimension
HRQoL: health-related quality of life
ICC: Intraclass Correlation Coefficient
KMO: Kaiser-Meyer-Oklin
LFI: Tucker-Lewis Index
LMICs: low-middle-income countries
NCDs: non-communicable diseases
PA: physical activity
PHQ-4: Patient Health Questionnaire-4
PLHIV: people living with HIV/AIDS
RMSEA: The Root Mean Square Error of Approximation
SD: standard deviation
SD: the coefficient of determination
SMD: standardized mean difference
SRMR: standardized root mean squared residual
SSA: Sub-Saharan Africa
