# Supplementary material for: Reliability and validity of the Shona version of the Exercise Benefits and Barriers Scale in Zimbabwean adult people living with HIV/AIDS
Source: Front Psychiatry. 2023 Aug 24;14:1188689. doi: 10.3389/fpsyt.2023.1188689 (PMC10483228; doi:10.3389/fpsyt.2023.1188689)
Supplement: Supplementary file 1 [file Data_Sheet_1.PDF]

Supplementary files

S1: Factor analysis plan

Exploratory factor analysis – we used the generalised least squares common factor analysis method. First, data were assessed for suitability for exploratory factor analysis. We tested/assessed the following factor pre-requisites: i) normality using the Shapiro Wilk Test {criterial value: p>0.05}, ii) the null hypothesis that the correlation matrix was different from a null matrix per Bartlett Test of Sphericity {criterial value: p<0.05} and iii) data was adequately spread across all response options i.e. it exhibited adequate sampling adequacy per Kaiser-Meyer-Oklin (KMO) measure {criterial value: KMO >0.7} [38].Using the Kaiser criterion, we retained factors with eigenvalues ≥ 1 were retained, and this was cross validated through inspecting the scree plot. Last. We applied oblique rotation (Promax method) to improve factors interpretability [38]. Given the conceptual differences, barriers and benefits were analysed separately.

Confirmatory factor analysis – we applied a non-recursive model constituted by the 26 items (manifest variables) on the EBBS-Shona and the hypothesised 6-factors (latent variables). We tested correlated and uncorrelated models, including the cross-validation of the exploratory factor analysis output. Outlined below are the criteria for goodness of fit [41]:

Fit statistic	Index	Criterion for fit
Likelihood Ratio	Chi-squared Test ($\chi ms2$)	p> 0.05
	$\chi 2/df$	<2
Population error	Root mean squared error of approximation (RMSEA)-(90% CI)	≤ 0.05
Information criteria	Akaike's information criterion (AIC)	Accept the model with the lowest AIC value
	Bayesian information criterion (BIC)	Accept the model with the lowest BIC value
Baseline comparison	Comparative fit index (CFI)	CFI≥0.90
	Tucker-Lewis index (LFI)	LT1≥0.90
Size of residuals	Standardised root mean squared residual (SRMR)	≤ 0.06
	The coefficient of determination (SD)	The greater the SD, the more useful the model

S2: EBBS-SF item descriptives

**Benefits items**			
**Item**	**Factor**	**Description**	**Mean (SD)**
EBBS1	PO	I enjoy exercise.	3.3 (.7)
EBBS2	PO	Exercise decreases feelings of stress and tension for me.	3.3 (.6)
EBBS3	PO	Exercise improves my mental health.	3.4 (.6)
EBBS6	PO	Exercising makes me feel relaxed.	3.3 (.6)
EBBS5	PP	Exercise increases my muscle strength.	3.2 (.7)
EBBS9	PP	Exercising increases my level of physical fitness.	3.4 (.5)
EBBS11	PP	My muscle tone is improved with exercise.	3.1(.6)
EBBS12	PP	Exercising improves my functioning cardiovascular system.	3.3 (.6)
EBBS13	PP	Exercise increases my stamina.	3.2 (.6)
EBBS26	PP	Exercise improves the way my body looks	3.3 (.6)
EBBS7	SI	Exercising lets me have contact with friends and persons I enjoy.	3.1 (.7)
EBBS18	SI	Exercising is a good way for me to meet new people.	3.1 (.7)
EBBS22	SI	Exercise is good entertainment for me.	3.2 (.6)
EBBS23	SI	Exercising increases my acceptance by others.	3.0 (.7)
EBBS15	LE	Exercising helps me sleep better at night.	3.3 (.6)
EBBS17	LE	Exercise helps me decrease fatigue.	3.0
EBBS19	LE	Exercising increases my mental alertness.	3.3
EBBS20	LE	Exercise allows me to carry out normal activities without becoming tired.	3.2 (.6)
EBBS24	LE	Exercise improves overall body functioning for me.	3.3 (.5)
Barriers items			
Item	Factor	Description	Mean (SD)
EBBS4	EM	Exercising takes too much of my time.	2.7 (SD.8)
EBBS10	EM	Exercise facilities do not have convenient schedules for me.	2.6 (.8)
EBBS16	EM	I think people in exercise clothes look funny.	2.9 (.7)
EBBS25	EM	There are too few places for me to exercise.	2.6 (.8)
EBBS8	TE	It costs too much to exercise.	2.9 (.7)
EBBS14	TE	Exercise takes too much time from family relationships.	2.8 (.7)
EBBS21	TE	Exercise takes too much time from my family responsibilities.	2.9 (.7)

Key: Benefits scales; LE- life enhancement, PP - physical performance, PO- psychological outlook, SI - social interaction. Barriers Subscales: EM - exercise milieu, TE - time expenditure

S3: EBBS-SF inter-item correlations

Benefits items

	Q1	Q2	Q3	Q6	Q5	Q9	Q11	Q12	Q13	Q26	Q7	Q18	Q22	Q23	Q15	Q17	Q19	Q20	Q24	**ITC**
Q1	1.000	0.551	0.425	0.377	-0.033	-0.062	0.025	0.004	-0.009	0.030	-0.001	-0.002	0.056	0.000	-0.088	0.013	-0.034	0.005	0.037	**0.209**
Q2	0.551	1.000	0.630	0.508	-0.014	0.029	0.052	0.035	-0.016	0.012	0.065	0.013	0.114	0.092	0.012	0.054	0.097	0.067	0.084	**0.302**
Q3	0.425	0.630	1.000	0.422	0.043	-0.033	0.026	0.016	-0.049	-0.018	0.081	-0.037	0.072	-0.002	-0.032	0.006	-0.005	-0.030	0.031	**0.226**
Q6	0.377	0.508	0.422	1.000	-0.006	0.030	0.064	0.036	-0.007	0.035	0.076	-0.040	-0.028	-0.026	-0.042	0.005	0.027	0.040	0.087	**0.225**
Q5	-0.033	-0.014	0.043	-0.006	1.000	0.450	0.510	0.439	0.358	0.384	0.415	0.348	0.390	0.222	0.290	0.269	0.316	0.347	0.349	**0.554**
Q9	-0.062	0.029	-0.033	0.030	0.450	1.000	0.500	0.522	0.454	0.480	0.506	0.402	0.358	0.300	0.463	0.413	0.448	0.413	0.559	**0.647**
Q11	0.025	0.052	0.026	0.064	0.510	0.500	1.000	0.527	0.502	0.479	0.495	0.475	0.416	0.386	0.409	0.437	0.474	0.460	0.507	**0.701**
Q12	0.004	0.035	0.016	0.036	0.439	0.522	0.527	1.000	0.599	0.510	0.542	0.453	0.514	0.370	0.541	0.402	0.573	0.489	0.565	**0.731**
Q13	-0.009	-0.016	-0.049	-0.007	0.358	0.454	0.502	0.599	1.000	0.482	0.558	0.597	0.430	0.464	0.579	0.468	0.557	0.479	0.528	**0.722**
Q26	0.030	0.012	-0.018	0.035	0.384	0.480	0.479	0.510	0.482	1.000	0.379	0.453	0.462	0.401	0.453	0.392	0.504	0.502	0.524	**0.669**
Q7	-0.001	0.065	0.081	0.076	0.415	0.506	0.495	0.542	0.558	0.379	1.000	0.581	0.430	0.473	0.429	0.348	0.495	0.433	0.490	**0.710**
Q18	-0.002	0.013	-0.037	-0.040	0.348	0.402	0.475	0.453	0.597	0.453	0.581	1.000	0.448	0.500	0.402	0.364	0.558	0.565	0.462	**0.690**
Q22	0.056	0.114	0.072	-0.028	0.390	0.358	0.416	0.514	0.430	0.462	0.430	0.448	1.000	0.484	0.429	0.367	0.583	0.468	0.498	**0.675**
Q23	0.000	0.092	-0.002	-0.026	0.222	0.300	0.386	0.370	0.464	0.401	0.473	0.500	0.484	1.000	0.398	0.291	0.506	0.342	0.393	**0.604**
Q15	-0.088	0.012	-0.032	-0.042	0.290	0.463	0.409	0.541	0.579	0.453	0.429	0.402	0.429	0.398	1.000	0.397	0.614	0.452	0.593	**0.651**
Q17	0.013	0.054	0.006	0.005	0.269	0.413	0.437	0.402	0.468	0.392	0.348	0.364	0.367	0.291	0.397	1.000	0.407	0.462	0.373	**0.593**
Q19	-0.034	0.097	-0.005	0.027	0.316	0.448	0.474	0.573	0.557	0.504	0.495	0.558	0.583	0.506	0.614	0.407	1.000	0.565	0.549	**0.740**
Q20	0.005	0.067	-0.030	0.040	0.347	0.413	0.460	0.489	0.479	0.502	0.433	0.565	0.468	0.342	0.452	0.462	0.565	1.000	0.581	**0.690**
Q24	0.037	0.084	0.031	0.087	0.349	0.559	0.507	0.565	0.528	0.524	0.490	0.462	0.498	0.393	0.593	0.373	0.549	0.581	1.000	**0.732**

Barriers items

	Q8	Q14	Q21	Q4	Q10	Q16	Q25	**ITC**
Q8	1	0.453	0.326	0.378	0.287	0.355	0.192	**0.667**
Q14	0.453	1	0.476	0.359	0.372	0.289	0.387	**0.746**
Q21	0.326	0.476	1	0.291	0.314	0.238	0.234	**0.642**
Q4	0.378	0.359	0.291	1	0.218	0.177	0.134	**0.583**
Q10	0.287	0.372	0.314	0.218	1	0.215	0.382	**0.650**
Q16	0.355	0.289	0.238	0.177	0.215	1	0.191	**0.555**

Q25	0.192	0.387	0.234	0.134	0.382	0.191	1													**0.575**

S4: Kaiser criterion for factor extraction

Barriers						
Factor	Initial Eigenvalues			Extraction Sums of Squared Loadings		
	Total	% of variance	Cumulative %	Total	% of variance	Cumulative %
1	2.829	40.413	40.413	2.226	31.803	31.803
2	.992	14.177	54.590			
3	.841	12.009	66.599			
4	.699	9.990	76.589			
5	.623	8.902	85.491			
6	.572	8.175	93.666			
7	.443	6.334	100.000			
Extraction Method: Generalised Least Squares.						

S5: Congenic CFA models for benefits and barriers factors

		**Likelihood Ratio**		**Population error**	**Information criteria**		**Baseline comparison**		**Size of residuals**	
**Category**	**Factor**	**χ2**	***χ2/df***	**RMSEA (95% CI)**	**AIC**	**BIC**	**CFI**	**LFI**	**SRMR**	**SD**
**Benefits**	LE	χ2 (df 5) =10.79, p<0.001	2.2	.064 (.000 - .117)	2193.2	2247.9	.989	.978	.024	.851
	PO	χ2 (df 2) =1.44, p<0.001	0.7	.000 (.000; .107)	1802.3	1846.1	1.000	1.004	.010	.833
	PP	χ2 (df 9) =18.64, p<0.001	2.1	.062 (.019; .101)	2678.0	2743.7	.985	.974	.029	.860
	SI	χ2 (df 2) =1.75, p<0.001	.9	.000 (.000;.113)	2117.6	2161.4	1.000	1.003	.015	.768
**Barriers**	EM	χ2 (df 2) =5.25, p<0.001	2.6	.076 (.000; .158)	2584.9	2628.6	.955	.864	.039	.692
	TE	χ2 (df 0) =0.000, p= _	_	.000 (.0:0.0)	1765.0	1797.8	1	1	0	.690

S6: EBBS item-total statistics, listwise deletion

Category	Factor	Item	Scale Mean if Item Deleted	Scale Variance if Item Deleted	Corrected Item-Total Correlation	Cronbach's Alpha if Item Deleted
Benefits	PO	EBBS1	73.43	62.359	.115	.857
	PO	EBBS2	73.46	61.613	.197	.855
	PO	EBBS3	73.42	61.919	.175	.855
	PO	EBBS6	73.46	61.959	.170	.855
Benefits	PP	EBBS5	73.60	57.801	.548	.844
	PP	EBBS9	73.40	58.736	.560	.844
	PP	EBBS11	73.64	57.700	.594	.843

	PP	EBBS12	73.49	58.286	.587	.843
	PP	EBBS13	73.57	58.016	.585	.843
	PP	EBBS26	73.49	58.734	.554	.845
Benefits	SI	EBBS7	73.68	57.035	.619	.841
	SI	EBBS18	73.73	57.448	.582	.843
	SI	EBBS22	73.55	58.994	.507	.846
	SI	EBBS23	73.76	58.256	.469	.846
Benefits	LE	EBBS15	73.48	58.897	.536	.845
	LE	EBBS17	73.74	58.011	.492	.845
	LE	EBBS19	73.49	58.017	.611	.843
	LE	EBBS20	73.59	58.532	.531	.845
	LE	EBBS24	73.47	58.737	.578	.844
	EM	EBBS4	74.50	60.487	.237	.855
Barriers	EM	EBBS10	74.33	60.002	.263	.854
	EM	EBBS16	74.68	61.469	.170	.857
	EM	EBBS25	74.32	60.898	.201	.856
Barriers	TE	EBBS8	74.69	60.364	.270	.853
	TE	EBBS14	74.59	60.211	.297	.852
	TE	EBBS21	74.60	61.660	.160	.857

S7: Comparison of EBBS scores by sex

	Males	Females	t-value	df	p-value
Psychological outlook Subtotal	13.8	13.1	3.59	565	0.000
Physical performance Subtotal	19.3	19.5	-0.63	565	0.529
Social Integration Subtotal	12.4	12.4	0.04	565	0.965
Life enhancement Subtotal	16.0	16.1	-0.39	565	0.699
Benefits Subtotal Score	61.4	61.0	0.64	565	0.520
Exercise milieu Subtotal	9.1	9.3	-0.78	565	0.438
Time expenditure Subtotal	6.4	6.4	0.17	565	0.867
Barriers Subtotal Score	15.6	15.7	-0.38	565	0.703
EBBS Total Score	77.0	76.7	0.40	565	0.687

S8: Correlations between EBBS and secondary variables

		TOTAL METs	Benefits Subtotal Score	EQ-5D Utility Score	PHQ-4 Total Score	Barriers Subtotal Score
TOTAL METs	r	1	.099*	-0.046	0.036	-0.043
	p-value		0.019	0.270	0.395	0.307
Benefits Subtotal Score	r	.099*	1	.094*	-.152**	-.118**
	p-value	0.019		0.026	0.000	0.005
Barriers Subtotal Score	r	-0.043	-.118**	0.043	-0.076	1
	p-value	0.307	0.005	0.307	0.071	
